# Pen-Based Graphomotor Measures for Early Identification of Attention-Related Difficulties in Preschool Children: A Conceptual and Critical Narrative Review

**DOI:** 10.3390/children13101375

**Published:** 2026-10-10

**Authors:** Muxian Ji, Tingshu Du, Tingting Ma, Luming Hu

**Affiliations:** 1 Bay Area School of Applied Psychological Sciences, Beijing Normal University at Zhuhai, Zhuhai 519087, Chinaduts@ion.ac.cn (T.D.);; 2 Faculty of Psychology, Beijing Normal University, Beijing 100875, China

**Keywords:** graphomotor assessment, digital handwriting, attention, preschool children, early identification, narrative review

## Abstract

**Highlights:**

**What are the main findings?**

- The available evidence suggests that attention-related variation may be reflected more clearly in the temporal organization of graphomotor behavior than in the completed graphic product. PGAM therefore organizes these temporal patterns into candidate regulatory components of engagement, maintenance, disruption, and recovery.
- Pen-based graphomotor measurement (PGAM) provides a traceable theory-to-measurement framework for testing links among controlled attentional demands, continuous pen trajectories, and temporally organized behavioral events.

**What are the implications of the main findings?**

- By reframing preliteracy graphomotor activity as an embodied perception–action context for attention measurement, PGAM offers a developmentally distinctive pathway for examining how goal-directed attentional regulation is expressed, destabilized, and restored before mature handwriting skills emerge.
- With rigorous developmental and psychometric validation, PGAM could support explainable, age-calibrated multimethod assessment for early risk stratification, targeted reassessment, and developmental monitoring.

**Abstract:**

Attention problems may compromise child development, learning, behavioral regulation, and school adjustment. The preschool years are an important period for early identification because attentional control develops rapidly and supports the transition to formal schooling. Existing assessment approaches, however, provide limited information about how attentional regulation unfolds during ongoing behavior. This non-systematic narrative review synthesizes evidence from preschool attention assessment, graphomotor development, digital handwriting, and measurement theory using a targeted, concept-driven approach. No systematic literature search or formal study-selection protocol was employed. It proposes pen-based graphomotor attention measurement (PGAM) as a measurement-development framework for examining how children select relevant information, maintain task goals, monitor movement, and correct deviations during graphomotor activity. PGAM links theoretically specified attentional demands and continuous pen-trajectory data to dynamic patterns of engagement, maintenance, disruption, and recovery during goal-directed behavior. Available evidence supports the technical feasibility of digital graphomotor recording and suggests associations between attention-related functioning and graphomotor organization. Future studies should first establish the reliability and developmental calibration of PGAM. Subsequent work should evaluate construct and incremental validity and decision utility to determine whether these process measures can support attention-specific interpretation and potential screening use.

## 1. Introduction

As preschool children navigate increasingly complex learning and social environments, attention helps them prioritize relevant information, sustain goal-directed behavior, resist distraction, and adapt to changing demands. Attention difficulties can compromise school readiness, educational attainment, social participation, and health-related quality of life [1,2,3]. The population burden is substantial, although estimates depend on definition and data source. An international meta-analysis estimated attention-deficit/hyperactivity disorder (ADHD) prevalence at 4.2–5.0%, with variation across sampling, informants, diagnostic procedures, and impairment criteria [4]. Separately, United States surveillance data showed that 11.4% of children aged 3–17 years had ever been diagnosed with ADHD by 2022, approximately one million more diagnosed children than in 2016 [5]. This surveillance statistic reflects cumulative diagnosed status and is therefore not directly comparable with the meta-analytic point-prevalence estimate. Although they represent different epidemiological indicators, both figures point to substantial service needs across families, education systems, and child health services and reinforce the value of timely identification and developmental support.

Attention difficulties are also relevant to developmental conditions beyond ADHD. A recent meta-analysis of 111 studies involving 12,292 children found small but significant attention delays across neurodevelopmental conditions during the first five years of life, although their magnitude varied by diagnosis, age, and measurement method [6]. The largest attention differences were observed in children with ADHD and communication disorders [6]. Preschool children with autism spectrum disorder also show atypical allocation of visual attention under specific stimulus conditions. Increased attention toward nonsocial material appears to be driven more strongly by objects related to restricted interests than by a general preference for all nonsocial stimuli [7]. Developmental dyslexia provides a further example of the relevance of attention to learning development. Two meta-analyses of visual attention span in developmental dyslexia [8,9] reported lower visual-attention-span performance in dyslexia, with effect sizes varying across task and sample characteristics, including stimulus type, writing system, age, and comparison group. Autism spectrum disorder and developmental dyslexia cannot be reduced to attention impairment. These findings nevertheless indicate that atypical attention can accompany several forms of developmental difficulty and can influence how children engage with social, linguistic, and educational information. Attention difficulties should therefore be identified early on the basis of their persistence, contextual pattern, and functional impact, allowing monitoring and support to begin before formal diagnosis is established.

The preschool years offer a critical window for identification. Expectations to follow instructions, sustain structured activity, complete goal-directed tasks, and participate in group learning make persistent difficulties increasingly visible. Meta-analytic evidence indicates that attention differences associated with neurodevelopmental conditions are difficult to identify reliably during infancy, begin to emerge during toddlerhood, and become more pronounced during the preschool years [6]. Marked preschool ADHD symptoms are associated with impairment in approaches to learning, social–emotional development, language, and motor functioning [2], while population-based longitudinal evidence links attention and activity problems at age three to poorer adolescent educational outcomes [3]. Identification before school entry can therefore support monitoring, family guidance, educational adjustment, and timely referral before classroom demands intensify.

### 1.1. Challenges and Limitations of Current Preschool Attention Assessment

Preschool screening must distinguish persistent developmental risk from ordinary variability. Short attention spans, variable engagement, and reliance on adult guidance are common at this age, and performance changes with interest, language comprehension, task familiarity, fatigue, environmental structure, and interpersonal support. Poor performance may arise from difficulty understanding instructions, tolerating testing, or producing the required response, without necessarily indicating a persistent attention problem. Mahone and Schneider [10] highlighted these challenges in preschool attention assessment, and more recent systematic evidence indicates that the diagnostic performance of ADHD tools still varies considerably across studies. A systematic review found that many diagnostic approaches show promising performance, but the overall strength of evidence is generally low and few studies provide estimates for children under seven years of age, indicating that preschool-specific validation remains limited [11]. Effective early screening therefore favors developmentally accessible, low-language methods that can be repeated in familiar settings and interpreted as part of a broader assessment.

Current assessment combines informant reports, direct behavioral measures, and digital or neurophysiological tools. Parent and teacher interviews and ratings capture behavior across home and educational settings, which is essential for identifying persistent cross-context difficulties. Their judgments also reflect observer expectations, situational demands, and developmental standards. In 452 children aged 3–5 years, parent-teacher agreement was low for both inattentive and hyperactive–impulsive symptoms [12]. A systematic review found stronger correspondence between teacher ratings and clinical interviews than between ratings and structured classroom observations, with higher validity estimates for hyperactivity-impulsivity than inattention [13]. Informant methods therefore provide indispensable contextual evidence, while process measures can add a direct record of how regulation unfolds during activity.

Direct observation and computerized attention tasks provide standardized evidence, but preschool performance still depends on emerging language, memory, cooperation, and motor skills. Poor performance may therefore reflect difficulty understanding instructions, retaining rules, tolerating testing, or producing the required response [10]. Structured observation captures attention within a defined activity, although conclusions remain specific to the setting, observer, and observation period. A 2025 review of 88 early-childhood studies found wide use of standardized and study-specific observation systems and emphasized alignment between the target behavior, observation method, and psychometric quality [14]. Continuous performance tasks quantify omissions, commissions, reaction time, and variability, with intra-individual reaction-time variability providing an established task-based marker of attentional instability, particularly in ADHD. Distributional measures such as ex-Gaussian τ characterize the contribution of infrequent, prolonged responses, whereas diffusion-model parameters provide model-based estimates of latent processes contributing to performance variability [15,16]. These approaches provide a refined characterization of instability across discrete responses, but standard summary indices do not directly characterize how instability unfolds within continuous goal-directed action, including its onset, persistence, task localization, correction, and recovery. Meta-analytic evidence shows modest-to-moderate discrimination for ADHD [17], and adding objective activity measures produces comparable performance [18]. These methods are most informative when integrated with broader assessment.

Digital and neurophysiological methods expand the range of observable signals, but their value for preschool screening depends on operational fit. Eye tracking measures visual orienting, yet data quality depends on calibration, positioning, operator skill, and management of movement and data loss [19]. Electroencephalography (EEG) in developmental research requires age-appropriate tasks, sufficient artifact-free trials, and specialized preprocessing. Movement artifacts, participant attrition, and analytical variation can reduce data quality and comparability [20]. Neuroimaging methods require specialized equipment, controlled testing environments, and careful management of motion, with these demands often limiting feasibility in young children [21]. These methods serve important research and selected clinical purposes, whereas broad preschool screening favors low-burden tools that can operate in familiar settings. Consistent with this distinction, a review of 231 diagnostic-tool studies found wide methodological variation and supported professional judgment based on evidence from multiple settings [11].

Together, these methods reveal a design opportunity. Preschool assessment needs ecological relevance, objective process data, developmental accessibility, and practical scalability in the same measurement context. Informant reports contribute cross-setting evidence, standardized tasks contribute controlled performance, and neurophysiological methods contribute detailed signals. Yet few approaches combine low burden, familiar task contexts, and time-resolved behavioral recording in a form suitable for repeated preschool assessment. In particular, established task-based variability measures characterize instability across discrete responses but provide a less direct account of how regulation destabilizes and is restored within an unfolding perception–action sequence. This creates a measurement gap for a process-sensitive method that can capture the temporal organization of regulation during goal-directed activity.

### 1.2. Pen-Based Graphomotor Measurement as a Process-Sensitive Approach

Pen-based graphomotor measures offer a promising solution to this need. Drawing, tracing, maze completion, and symbol copying are familiar preschool activities that require children to sustain goal-directed engagement while continuously coordinating hand movements with an external visual goal. When these activities are completed using a digitizing tablet, pressure-sensitive stylus, smart pen, or instrumented writing surface, the final graphic product can be supplemented by high-resolution temporal, spatial, and kinematic data. Candidate measures span movement initiation and pausing, kinematic and pressure characteristics, spatial deviation, and corrective behavior. These measures preserve information about the temporal progression and stability of performance that is lost when assessment is restricted to completion time, error count, or final accuracy. Digital recording therefore extends conventional graphomotor assessment beyond the completed product by preserving a time-resolved record of how behavior unfolds during task performance.

Research in adjacent graphomotor fields establishes the technical feasibility of this approach. Dui et al. [22] demonstrated that fundamental graphomotor principles could be quantified through symbol drawing before formal handwriting acquisition. Their tablet-based application captured developmental variation in movement time, spatial scaling, and the relation between movement speed and accuracy in kindergarten children. Asselborn et al. [23] extracted 53 handwriting features from 298 children and showed that movement characteristics recorded using a consumer tablet could distinguish children with and without dysgraphia. A systematic review of handwriting in children with ADHD also identified recurrent differences in handwriting products and dynamic processes, including temporal organization, pen pressure, and movement fluency [24]. These findings form part of a broader process-oriented tradition in handwriting research. Digitizer-based process analyses distinguish the completed product from the temporal and kinematic organization of its production, while Eye and Pen approaches examine the coordination of visual exploration with ongoing writing. Digital BHK (Concise Assessment Scale for Children’s Handwriting) methods complement product-based scoring with dynamic measures, and tablet-based studies have extended process characterization to multidimensional temporal, kinematic, pressure, and spatial feature sets. The Handwriting Proficiency Screening Questionnaire (HPSQ) and Handwriting Proficiency Screening Questionnaire for Children (HPSQ-C) provide a complementary perspective by assessing perceived handwriting difficulties through teacher report or child self-report without continuous trajectory recording [23,25,26,27,28,29,30,31,32]. Together, these approaches provide a well-developed methodological basis for characterizing graphomotor performance beyond its final graphic outcome. More broadly, continuous graphomotor data can be understood as process-level behavioral traces generated as cognitive, perceptual, and motor activity unfolds over time. Their temporal structure can be transformed into behaviorally meaningful events, such as initiation, pausing, deviation, and correction. What remains less clear is how these observable events map onto the cognitive processes operating during task performance.

Building on this process-oriented foundation, we propose pen-based graphomotor attention measurement (PGAM) as a measurement-development framework for linking graphomotor process data to theoretically defined aspects of attentional regulation in preschool children. PGAM organizes established process signals according to their hypothesized relation to changes in attentional regulation during goal-directed graphomotor activity, without introducing a new class of trajectory variables. Its principal extension lies in mapping the temporal organization of observable graphomotor events onto the cognitive regulation they may reflect, thereby providing a testable basis for attention-related interpretation. The position of PGAM relative to established handwriting and graphomotor assessment approaches is summarized in Table 1.

### 1.3. Objectives and Scope of the Present Review

The central scientific question is whether attention-related variation can be identified within graphomotor behavior while distinguishing attentional contributions from the perceptual, motor, and developmental processes that jointly shape performance. Addressing this question requires a theoretical account of how attention may become observable in continuous graphomotor behavior and a reproducible framework that translates this account into testable measurement operations. PGAM is proposed as this measurement architecture, with the developmental graphomotor account providing its theoretical link between established attention theory and continuous graphomotor behavior.

The present conceptual and critical narrative review therefore examines how attentional regulation may be expressed in pen-based graphomotor behavior, how these processes can be operationalized through task design and process measures, and what psychometric evidence is required to support their interpretation. It further considers the potential role of this approach in early identification, developmental monitoring, and subsequent support. Pen-based graphomotor measurement is positioned as a complementary component of multisource assessment, with diagnostic interpretation reserved for comprehensive evaluation.

## 2. Review Approach

This article adopts a conceptual and critical narrative review approach. It integrates evidence from four domains: preschool attention assessment, graphomotor development, digital handwriting and graphomotor measurement, and contemporary validity theory. Literature was synthesized according to its relevance to the proposed measurement-development argument. Direct evidence from preschool children is prioritized when available, while evidence from school-aged, adolescent, adult, or clinical samples was used to clarify technical feasibility, plausible mechanisms, competing explanations, or validation requirements. The purpose is to organize a testable validity argument, not to estimate a pooled effect or provide exhaustive coverage of the literature.

No preregistered protocol, reproducible database search, dual-reviewer study selection, or formal risk-of-bias assessment was conducted. Accordingly, this article is a non-systematic narrative review and does not claim systematic or scoping-review coverage. Evidence is organized according to its role in evaluating PGAM. Digital handwriting and graphomotor studies establish technical measurability, while developmental evidence addresses the interpretability of these measures during the preschool period. Controlled task studies and associations with independent attention measures inform attention-related construct interpretation, including discrimination from perceptual and motor influences. Representative preschool studies are ultimately required to establish screening accuracy and decision utility. Together, these evidential roles structure the validity argument developed for PGAM.

## 3. Theoretical Foundations of Pen-Based Graphomotor Attention Measurement

PGAM rests on three propositions. First, graphomotor activity is continuous and goal-directed, so its temporal organization can express how children select information, engage with a task, maintain a goal, monitor movement, and adjust behavior. Second, attention and graphomotor coordination develop rapidly during the preschool years. Limited motor automaticity may increase the behavioral visibility of regulatory variation during this period. Third, digital recording preserves the trajectory information needed to characterize engagement, maintenance, disruption, and recovery. PGAM therefore shifts assessment from static products to the time-resolved organization of goal-directed behavior.

### 3.1. Graphomotor Activity as a Continuous Behavioral Expression of Attention

Graphomotor activity links an external visual goal to an unfolding motor response. Children identify a target, select a path, execute movement, monitor feedback, and correct deviations. Van Galen’s hierarchical model described how cognitive representations are transformed into motor commands, while Danna and Velay distinguished advance preparation from online control through visual, proprioceptive, and kinesthetic feedback [34,35]. Although developed for handwriting, both accounts show that a visible trajectory emerges from coordinated cognitive, perceptual, and motor processes. Its temporal structure may therefore preserve information about how regulation unfolds during action.

The developmental graphomotor account underlying PGAM draws on established attention-network models that distinguish alerting, orienting, and executive control, together with developmental work on sustained attention and reorientation after distraction [36,37,38,39]. Alerting supports readiness to respond, orienting prioritizes task-relevant information, sustained attention supports continued goal maintenance, and executive control contributes when distraction, conflict, or changing task demands must be resolved. These attentional functions provide the theoretical basis for specifying the task demands examined within PGAM. PGAM does not assume that these attentional functions can be read directly from pen movement. Instead, it examines whether changes in the temporal organization of goal-directed graphomotor behavior provide candidate expressions of attentional regulation that can be validated through controlled task manipulations. A completed graphic product may reveal a boundary crossing, omission, or malformed shape, whereas continuous recording preserves the sequence through which such regulatory changes unfold.

Available evidence supports an association between attention and the organization of graphomotor behavior. In a study of 867 primary-school children, selective attention predicted handwriting fluency after spelling and graphomotor proficiency were taken into account, but showed no comparable independent association with legibility. This pattern suggests that attentional contributions may be more evident in the continuity and efficiency of production than in the static appearance of the final trace [40]. Longitudinal evidence spanning kindergarten to first grade similarly showed that kindergarten inattention predicted later Chinese-character accuracy and construction, whereas manual dexterity primarily predicted writing speed and visual–motor integration predicted character construction [41]. A systematic review of handwriting in children with ADHD further identified potential differences in contact and non-contact duration, pressure, velocity, velocity peaks, and fluency [24]. Collectively, these findings support the plausibility of an attention–graphomotor association, but they do not establish attention specificity for any individual trajectory feature.

Graphomotor performance emerges from the joint influence of attentional, perceptual, motor, motivational, and task-related factors. Within PGAM, attention-related regulation is therefore treated as one contributor to how children select task-relevant information, maintain goals, and adjust ongoing behavior. Continuous trajectories are not interpreted as direct records of attention. Their attention-related meaning must instead be established through controlled attentional manipulations and empirical discrimination from competing perceptual, motor, developmental, and contextual explanations.

### 3.2. Developmental Sensitivity of Graphomotor Activity in Preschool Children

The preschool years are characterized by concurrent development in attentional control and graphomotor competence. Children become increasingly able to select task-relevant information, maintain behavioral goals, and re-engage following distraction, although these capacities remain variable and incompletely stabilized. A meta-analysis found that attention differences associated with neurodevelopmental conditions were not reliably detectable during infancy, emerged during toddlerhood, and became more pronounced during the preschool period [6]. The preschool years therefore represent a developmental interval in which meaningful attention-related differences become increasingly detectable and require developmentally appropriate measurement.

The internal organization of attention also becomes more differentiated during this period. Deodhar and Bertenthal [42] found that attentional control in preschool children was closely related to executive functioning but was nevertheless represented by a distinguishable latent factor. Kim et al. [39] further decomposed selective sustained attention into the capacity to remain engaged with a target and the capacity to return after distraction. Between 3.5 and 6 years of age, the ability to return improved substantially and contributed importantly to developmental gains in selective sustained attention. Children may therefore display similar brief interruptions while differing markedly in reorientation speed and subsequent stability. Continuous graphomotor behavior provides a plausible context in which these temporally distinct patterns can become observable.

Graphomotor control during the preschool years remains strongly dependent on sensory feedback and online adjustment. Children can generate purposeful trajectories, but target localization, movement direction, speed regulation, force control, and spatial correction are not yet supported by the highly automated motor programs characteristic of experienced writers. Guilbert et al. [43] found that reductions in visual and proprioceptive feedback had stronger effects on handwriting in children than in adults, altering movement speed, letter size, and legibility. Although the children in that study were older than preschool age, the developmental comparison indicates that less experienced writers depend more heavily on continuously available information. Transient variation in attention may therefore be more likely to influence movement initiation, pause organization, trajectory stability, and corrective behavior when graphomotor control remains effortful and feedback dependent.

Studies conducted directly with preschool children show that graphomotor behavior is already sufficiently structured for quantitative analysis. Dui et al. [22] identified systematic relations among movement amplitude, duration, velocity, and accuracy during symbol drawing before formal handwriting acquisition, with these relations continuing to develop with age. Chu and Krishnan [44] quantified multiple dimensions of finger-based pattern copying on a tablet, whereas No and Choi [45] found that paper and tablet conditions systematically affected movement size, speed, pressure, and graphic clarity in six-year-old children. A video-coded study of children aged 3 to 6 years who copied and then reproduced a complex figure found that even the youngest children oriented primarily to the task, that gaze targets changed over intervals of only a few seconds, and that gaze organization varied with performance level [46]. In a community sample of 611 children aged 3 to 6 years, Gilboa [47] reported test–retest reliability of 0.95 and interrater reliability of 0.94 for the Gilboa Functional Test, a norm-referenced graphomotor screening measure, together with modest associations with visual–motor integration and manual dexterity. Preschool graphomotor activity is therefore neither random movement nor a reduced form of mature handwriting. It is a structured, developmentally sensitive, and actively regulated behavioral system. Its limited automatization may increase the visibility of attention-related variation, while also requiring perceptual–motor development and recording conditions to be considered in interpretation.

### 3.3. Dynamic Components of Goal-Directed Regulation in PGAM

The theoretical basis for PGAM distinguishes established attentional functions from the dynamic organization of goal-directed behavior. Alerting, orienting, sustained attention, and executive control/reorientation provide the attentional mechanisms of interest [36,37,38,39,48]. PGAM does not assume that these functions can be read directly from pen movement. Instead, it examines how their influence may become observable through changes in the organization of continuous graphomotor behavior.

Engagement, maintenance, disruption, and recovery therefore capture complementary temporal dynamics as goal-directed regulation unfolds. Engagement refers to the establishment of functional alignment between the task goal and ongoing action. Maintenance refers to the stability of this goal-directed organization over time. Disruption denotes a transient loss of stable goal–behavior coordination, whereas recovery refers to the re-establishment of stable goal-directed regulation following interruption or deviation. Established attentional functions may contribute to more than one of these components, and each component may reflect multiple cognitive, perceptual, and motor influences.

Process-sensitive measurement requires preservation of the temporal structure through which a graphic trace is produced. Paper-and-pencil assessment retains the final product but not the pauses, deviations, corrections, or returns to stable performance that shaped it. Similar products may arise from stable performance or repeated interruption and adjustment. Digital tablets, instrumented pens, and electronic writing surfaces preserve coordinates, time, contact, pressure, velocity, and direction, allowing graphomotor behavior to be reconstructed as a continuous process.

Digital prewriting research indicates that such recording is feasible before mature handwriting has developed. Dui et al. [22] quantified isochrony, homothety, and speed–accuracy relations during symbol production at the preliteracy stage. Chu and Krishnan [44] extracted multiple dimensions from finger-based pattern copying on a tablet and found preliminary group differences between typically developing children and children receiving occupational therapy for visual–motor difficulties. No and Choi [45] recorded movement size, speed, and pressure in preschool children across tablet and paper conditions. These studies establish the digital measurability of preschool graphomotor behavior, although they do not demonstrate that the resulting variables provide attention-specific information.

Within this account, PGAM organizes continuous trajectories around four dynamic regulatory components: engagement, maintenance, disruption, and recovery. These components are not direct measures of attention. Rather, they provide a dynamic organizational framework for testing how experimentally defined attentional demands become expressed in ongoing behavior. The mapping is deliberately many-to-many: established attentional functions may influence more than one regulatory component, and each behavioral expression must be interpreted in relation to task demands, developmental characteristics, and competing perceptual and motor explanations [39].

These components form a dynamic and potentially recurrent organization of goal-directed regulation and should not be interpreted as a fixed sequence of task phases. One child may engage rapidly but become progressively unstable, whereas another may briefly deviate and then efficiently restore the preceding movement pattern. Overall accuracy and completion time may fail to distinguish these forms of regulation, while continuous trajectories preserve their sequence and temporal evolution. PGAM therefore targets the continuous organization of graphomotor regulation, not general handwriting quality or isolated movement parameters.

**Figure 1:**
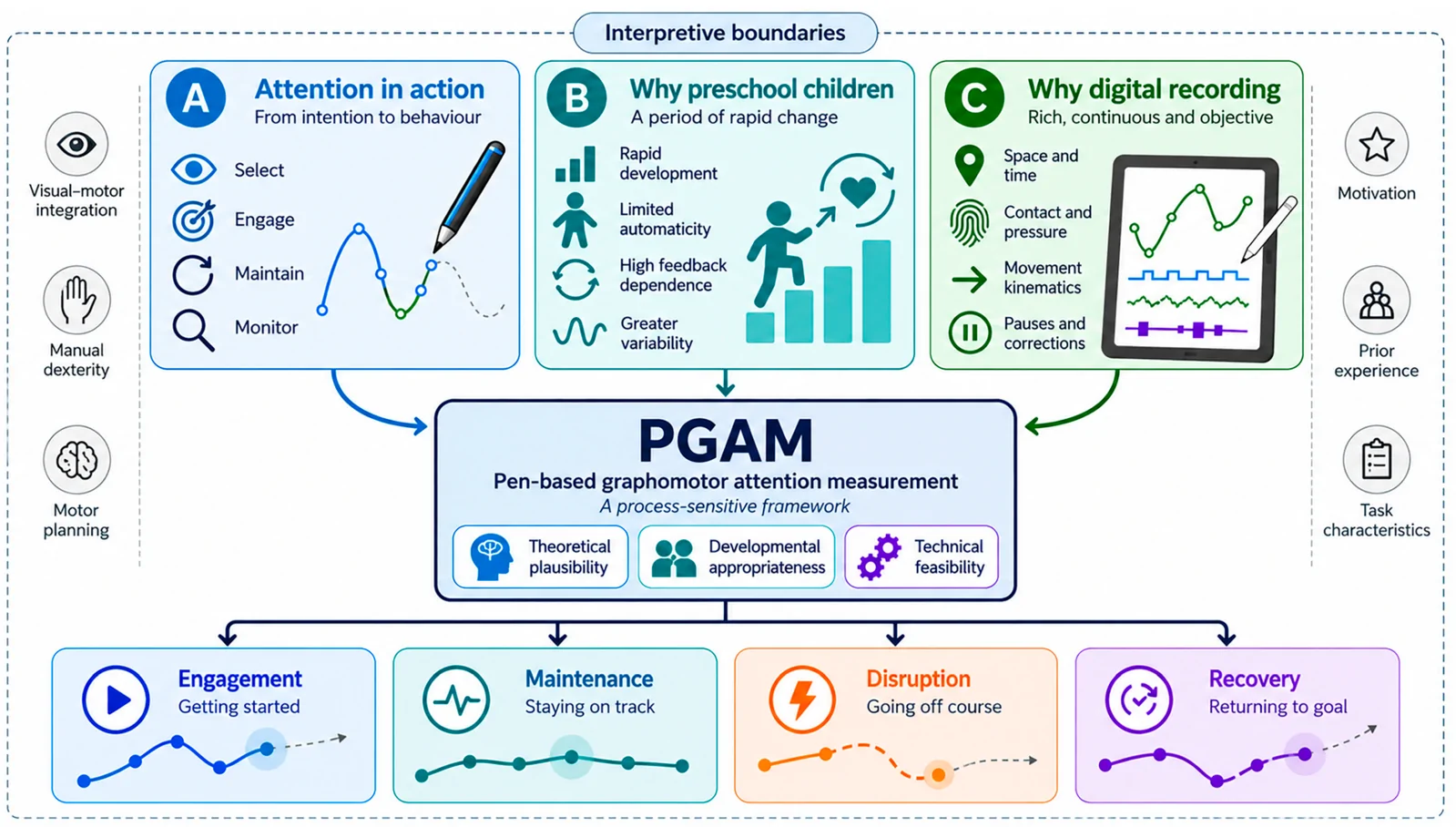
Conceptual organization and interpretive boundaries of pen-based graphomotor attention measurement (PGAM). Note: PGAM integrates graphomotor activity, preschool developmental characteristics, and digital recording into candidate regulatory components of engagement, maintenance, disruption, and recovery. These components are not direct measures of attention and should be interpreted within relevant perceptual, motor, motivational, experiential, task, and device-related boundaries. PGAM = pen-based graphomotor attention measurement.

PGAM draws strength from three converging foundations. Graphomotor activity provides a continuous context in which attentional regulation can be expressed. Preschool motor control remains effortful and feedback dependent, increasing the visibility of regulatory variation. Digital recording preserves the temporal and spatial structure needed to characterize these regulatory dynamics. Figure 1 brings these foundations together as a theoretically grounded, developmentally appropriate, and technically feasible framework whose indicators can be tested against explicit attention and screening criteria.

### 3.4. Evidence Integration and Construct Boundaries of Graphomotor Indicators

The evidence supports a differentiated account of pen-based graphomotor measurement. Technical measurability is well established, while several studies suggest associations between attention-related functioning and the temporal organization of graphomotor behavior. Table 2 shows how studies across ages, clinical groups, writing systems, tasks, and devices contribute distinct parts of the PGAM rationale. This diversity defines the validation agenda: preschool studies must connect controlled attentional demands to reproducible trajectory patterns and screening-relevant interpretation.

Evidence for technical measurability is the most consistent. Graphomotor production requires the continuous coordination of movement planning, motor execution, sensory feedback, and online correction, making the evolving trajectory a plausible source of process-level information beyond the final graphic product [34,35]. Digital tablets and instrumented pens can preserve spatial coordinates, contact states, pressure, velocity, directional changes, pauses, and corrective movements at sufficient temporal resolution to reconstruct the production process. Studies conducted before formal handwriting acquisition further indicate that preschool-aged children can complete structured drawing, tracing, and pattern-copying tasks while generating quantifiable and analyzable movement records [22,44,45]. Collectively, these findings establish the feasibility of continuous graphomotor measurement during the preschool period. They do not, however, identify which recorded features are specifically attributable to attention or whether such features are sufficiently reliable and valid to support individual-level screening decisions.

Evidence linking attention to graphomotor organization is recurrent but heterogeneous. Selective attention appears to predict handwriting fluency more consistently than final legibility, suggesting that attentional contributions may be expressed more clearly in the continuity and regulation of production than in the static appearance of the completed trace [40]. Studies of children with ADHD have also reported differences in writing-size consistency, non-contact duration, speed or pressure variability, fluency, and graphic quality [24,49,50]. These findings do not converge on a single indicator or a consistent direction of effect. Medication studies further suggest that improvements in behavioral control and graphic quality may coexist with reduced kinematic fluency, whereas some unmedicated samples show little difference from typically developing children in mean fluency [51,52]. Attention-related regulation, motor automaticity, and graphic quality should therefore be regarded as associated but analytically distinguishable components of performance. Temporally localized patterns of variability, deviation, correction, and recovery may offer greater interpretive value than isolated mean values, although this proposition requires direct empirical testing.

The central unresolved issue is construct attribution. Perceptual and motor abilities, task comprehension, motivation, and prior graphomotor experience can influence many of the same trajectory characteristics hypothesized to vary with attention. Evidence from Chinese handwriting research suggests that motor and visual–motor abilities may account more directly for legibility and production time than attention alone [53]. Longitudinal findings similarly indicate that inattention, manual dexterity, and visual–motor integration predict partly distinct aspects of subsequent handwriting performance [41]. Moreover, test–retest stability varies across preschool handwriting measures [54]. Evidence from primary-school children further indicates that, even when the factor structure and factor loadings of a handwriting-legibility scale are comparable across grades, intercept differences may limit direct comparisons of mean scores between some grade groups [55]. A study comparing 41 preschool children with 40 young adults found executive-motor associations, particularly those involving working memory, in preschoolers but not in young adults [56]. This preliminary evidence suggests that cognitive and motor demands may be more closely intertwined during early graphomotor development. An atypical trajectory characteristic therefore cannot be interpreted as direct evidence of an attention problem without accounting for alternative perceptual, motor, developmental, and contextual explanations.

Motor coordination should therefore be treated as a competing source of observed variance and a direct threat to construct attribution, with covariate adjustment used as a supplementary control within a broader construct-validation strategy. This issue is especially important because ADHD and developmental coordination disorder (DCD) frequently co-occur, with a recent review estimating substantial overlap between the two conditions [57]. International clinical recommendations for DCD also emphasize the need to assess motor coordination difficulties directly and to consider co-occurring neurodevelopmental conditions when interpreting functional performance [58]. The evidence summarized in Table 2 illustrates this problem: the Chinese handwriting study included children with ADHD without DCD, yet motor skill and visual–motor integration were still associated with handwriting legibility and temporal measures [53]. Excluding DCD therefore does not eliminate motor-related ambiguity in the interpretation of graphomotor indicators. Statistical adjustment for motor variables alone is likewise insufficient to establish attention specificity.

Concurrent standardized assessment of motor coordination and visual–motor integration, together with baseline graphomotor proficiency, should therefore form a core component of PGAM construct validation. Clinical validation studies intended to establish attention specificity should, where appropriate to the study design, include typically developing, ADHD-only, DCD-only, and ADHD+DCD comparison groups so that attentional, motor, and comorbid contributions can be evaluated directly.

Computational studies reinforce the distinction between discriminative performance and construct validity. High-dimensional trajectory features can classify dysgraphia or handwriting difficulty with substantial accuracy in selected samples, but these models target graphomotor impairment and do not directly assess attentional regulation [23,59]. In a sample of 305 children, including 43 with dysgraphia, Devillaine et al. [59] reported a maximum cross-validated classification accuracy of approximately 73%. Gargot et al. [27] found that three of 12 digital features—two kinematic and one static—were significantly associated with handwriting-quality variation among typically developing children. These findings demonstrate the discriminative and descriptive potential of digital trajectory data. They do not, by themselves, establish the psychological meaning, developmental stability, attention specificity, or practical screening value of a signal.

**Table 2:** Critical Evidence Map for the Theoretical and Measurement Foundations of Pen-Based Graphomotor Attention Measurement.

Evidence Domain	Representative Study and Design	Principal Finding	Contribution to PGAM	Boundary of Inference
Theoretical	Theoretical psychomotor model [35]	Handwriting was conceptualized as a hierarchical system in which cognitive, psychomotor, and biophysical processes interact to generate observable writing movements.	Establishes the theoretical rationale for examining the unfolding production process, extending analysis beyond the completed graphic product.	The model explains handwriting production but does not specify attention-related indicators, developmental screening criteria, or individual-level decision rules.
Attention–graphomotor association	Cross-sectional multigroup structural equation modeling study of 867 children in Years 3–5 [40]	Selective attention predicted handwriting fluency, but not legibility, after graphomotor and spelling skills were modeled. It also partially mediated the association between graphomotor skill and fluency.	Provides comparatively direct evidence that attentional contributions may be expressed more clearly in production continuity than in final graphic appearance.	The study was limited to school-aged children and conventional handwriting outcomes and did not assess preschool digital trajectories or attention-screening performance.
Attention–graphomotor association	Systematic review of 21 articles, including 14 examining handwriting-process measures [24]	Among studies meeting satisfactory methodological criteria, handwriting-quality differences were reported in 11 of 11 studies, process abnormalities in 11 of 12, and speed differences in 2 of 8.	Supports a recurrent association between ADHD and graphomotor organization while indicating that multidimensional process patterns are more informative than production speed alone.	The evidence was heterogeneous in age, medication, comorbidity, ADHD presentation, task structure, and outcome definition and does not identify attention-specific preschool indicators.
Attention–graphomotor association	Case–control digitizer study of 28 boys, including 14 with ADHD-combined type [49]	Boys with ADHD showed greater inconsistency in writing size, and greater symptom severity, particularly inattention, predicted poorer handwriting outcomes.	Identifies within-production inconsistency as a plausible correlate of attention-related graphomotor dysregulation.	The small, all-male sample and repeated cursive-letter task limit developmental, task, and population generalizability.
Attention–graphomotor association	Case–control digitizer study of 21 children with ADHD without developmental coordination disorder and 21 matched controls [53]	Children with ADHD produced less legible writing and required more on-paper and total writing time. Motor skill and visual–motor integration were associated with legibility, while motor skill was also associated with temporal measures.	Shows that motor and visual–motor factors contribute to graphomotor performance even in children with ADHD without DCD, underscoring the need to distinguish attentional from motor sources of variation.	Excluding DCD reduces one major source of motor comorbidity but does not eliminate motor-related ambiguity. The study was cross-sectional and did not establish the attention-specific incremental validity of digital graphomotor indicators.
Attention–graphomotor association	Digitizer medication studies with child, adult, and control comparisons [51,52]	Methylphenidate improved aspects of handwriting quality while reducing kinematic fluency. Under constrained tasks, unmedicated participants with ADHD did not consistently differ from controls in mean fluency.	Shows that product quality, behavioral control, and motor automaticity are distinguishable and that mean fluency is not a direct marker of attentional impairment.	Samples were small, tasks involved highly practiced cursive movements, and medication and task instructions substantially influenced performance.
Attention–graphomotor association	Matched case–control digitizer study of 16 children with ADHD and 16 age- and sex-matched controls [60]	Across figure-copying tasks, children with ADHD produced slower, less accurate strokes with higher axial pen force. The findings did not support impaired motor planning but indicated less effective parameter setting as accuracy demands increased.	Shows that digitized graphic movements are sensitive to task constraints and that observed group differences may reflect motor-control processes that overlap with, but are not equivalent to, attention.	The sample was small and school-aged, and the study did not evaluate preschool children, attention-specific indicators, or screening accuracy.
Construct validity	Review of ADHD–DCD co-occurrence and international clinical recommendations for DCD [57,58]	ADHD and DCD frequently co-occur, and clinical recommendations emphasize direct assessment of motor coordination difficulties and consideration of co-occurring neurodevelopmental conditions.	Establishes motor coordination as a core construct-validity consideration when graphomotor output is used to examine attention-related regulation.	These sources establish the relevance of motor contributions but do not determine the attention specificity of PGAM indicators or the optimal validation design.
Developmental	One-year longitudinal observational study of 106 kindergarten children [41]	Kindergarten inattention predicted later character accuracy and construction, whereas manual dexterity primarily predicted writing speed and visual–motor integration contributed to character construction.	Provides developmentally relevant evidence that attentional, motor, and visual–motor abilities predict partly distinct graphomotor outcomes.	Inattention was assessed using behavioral ratings, and the study focused on later handwriting outcomes without examining concurrent preschool digital attention indicators.
Developmental	Tablet-app validation study involving 19 kindergartners and 15 third-grade children [22]	Isochrony, homothety, and speed–accuracy principles were observable during tablet-based symbol drawing before formal handwriting acquisition, with several measures differing by age.	Establishes that developmentally appropriate digital tasks can capture quantifiable graphomotor processes during the preliteracy period.	The samples were small and typically developing, and the study addressed graphomotor principles without evaluating attentional regulation or screening validity.
Developmental	Instrument-development study of 482 preschool children with a test–retest subsample of 27 [54]	The HT-PRE showed acceptable internal consistency, but test–retest coefficients varied substantially across subtests. Scores were associated with visual–motor integration and differentiated normative and handwriting-difficulty groups.	Demonstrates the feasibility of structured preschool graphomotor assessment and highlights the importance of indicator-level stability.	The measure was primarily paper based and product oriented, the retest sample was small, and several subtests showed limited temporal stability.
Developmental	Scale-development and validation study of 684 children in Grades 2, 4, and 6 [55]	The handwriting-legibility scale supported configural and metric invariance across grades but did not demonstrate full scalar invariance because of intercept differences between Grades 2 and 4.	Shows that reliable graphomotor scores may preserve a common structure while differing in reference level across developmental groups.	The measure assessed product-level legibility in school-aged children and does not establish developmental equivalence of continuous PGAM indicators.
Technical	Matched group-comparison digitizer study of 100 third-grade children, including 50 proficient and 50 dysgraphic writers [33]	Total, on-paper, and in-air time and path-length measures differentiated proficient and dysgraphic writers across copying and dictation tasks. Process and product measures provided complementary information.	Demonstrates that continuous recording preserves temporal and spatial information unavailable from the completed product alone.	The findings concern developmental dysgraphia in school-aged children and provide no direct evidence on attentional regulation or preschool screening.
Technical	Multidimensional digital-handwriting study of 280 children [27]	Only three of 12 digital features were significantly associated with handwriting-quality variation among typically developing children, and several features showed age-related interactions.	Supports theory-guided indicator construction and developmental calibration, with device-available variables interpreted only within prespecified theoretical and developmental constraints.	The criterion was handwriting quality rather than attention, and developmental patterns were estimated cross-sectionally.
Technical	Machine-learning studies of tablet-recorded handwriting and graphomotor tasks [23,59]	Asselborn et al. reported an F1 score of 97.98% in 298 children, but this estimate was obtained through internal cross-validation and used dysgraphia labels derived from the BHK handwriting-quality assessment, absent any independent clinical or screening outcomes. Devillaine et al. obtained a maximum cross-validated accuracy of approximately 73% in a more heterogeneous database of 305 children.	Demonstrates the computational feasibility of automated graphomotor classification while showing that estimated performance depends strongly on task design, sampling, feature construction, and validation strategy.	Both studies targeted dysgraphia and relied on internal validation. Neither established independent external validity, calibration, transportability, or attention specificity.

Note: Studies of handwriting, dysgraphia, ADHD, and DCD are included as indirect evidence relevant to the theoretical, developmental, and technical foundations and construct boundaries of PGAM. ADHD = attention-deficit/hyperactivity disorder; HT-PRE = Handwriting Test for Preschool Children; PGAM = pen-based graphomotor attention measurement.

## 4. Technical Framework for Pen-Based Graphomotor Attention Measurement

**Figure 2:**
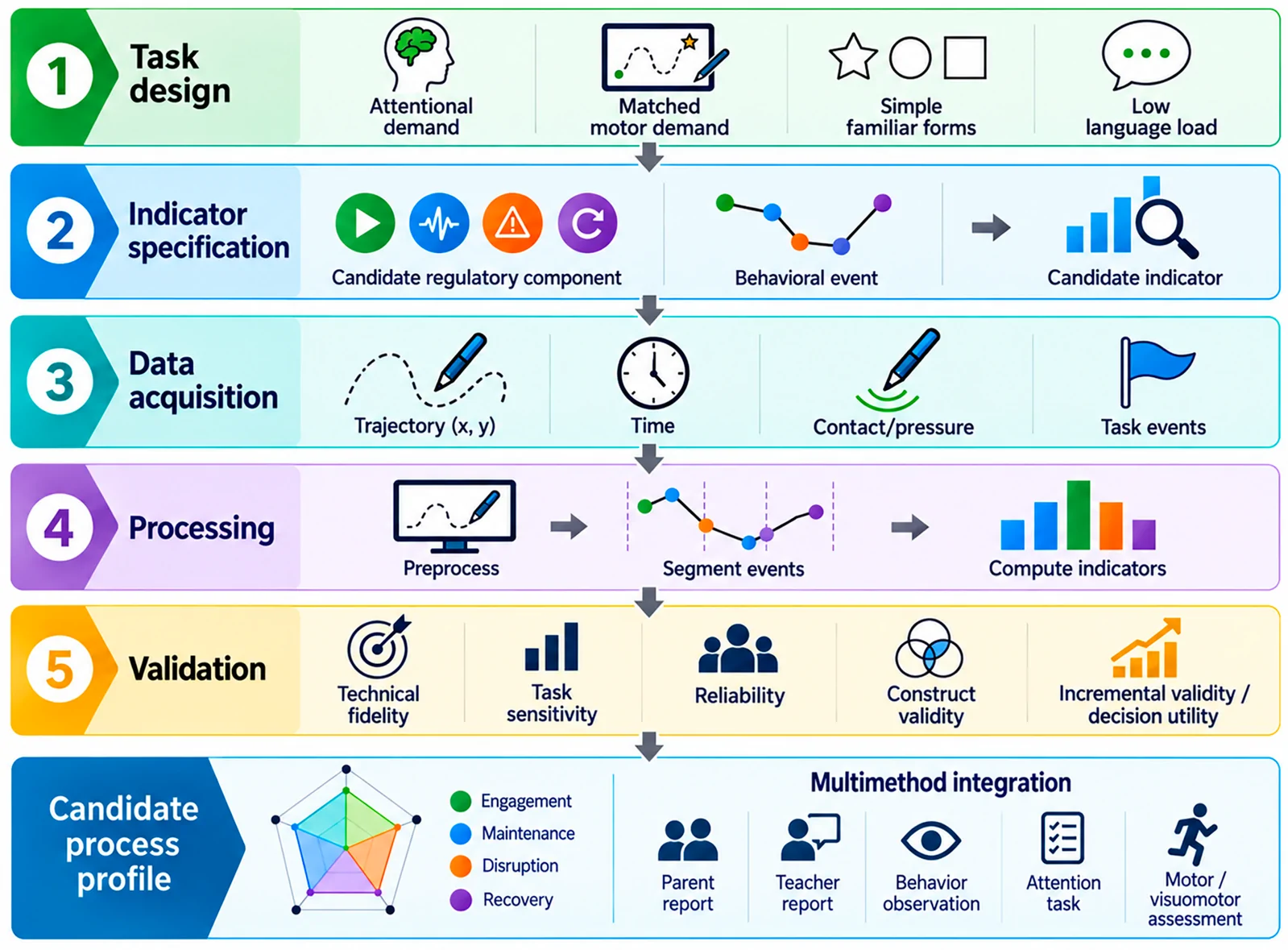
Technical architecture and validation pathway of pen-based graphomotor attention measurement (PGAM). Note: The PGAM architecture links task design, indicator specification, data acquisition, processing, and validation. Candidate indicators representing engagement, maintenance, disruption, and recovery may contribute to a process profile only after adequate validation and should be interpreted within multimethod attention assessment. PGAM = pen-based graphomotor attention measurement. Arrows represent conceptual relationships rather than empirically established causal pathways.

PGAM becomes empirically testable when its theoretical propositions are translated into explicit measurement operations. A PGAM research program would therefore require an integrated technical architecture (Figure 2). This architecture links theory-guided task design and a priori indicator specification with standardized acquisition, reproducible processing, candidate-indicator construction, and psychometric evaluation. Task design would create distinguishable attentional demands while controlling motor and graphic requirements. Indicator specification would define expected behavioral events and candidate measures before data analysis. Standardized acquisition would preserve temporal structure, whereas preprocessing and event segmentation would transform raw records into reproducibly computed candidate indicators. Psychometric evaluation would then determine which indicators are interpretable, technically stable, and externally replicable. Validation findings should feed back into task and indicator refinement, making the analytical pipeline iterative.

### 4.1. Task Design for Interpretable Attentional Demands

PGAM task design begins with a defined attentional process and embeds it in a controlled graphomotor activity. Tracing, maze completion, symbol connection, and figure copying all require attention, but they differ in visual encoding, spatial organization, sequential maintenance, and motor control. Completion time or overall accuracy alone cannot identify the source of trajectory differences. Graphic properties and response rules should therefore isolate the intended psychological demand while holding competing motor demands as constant as possible.

Tasks for preschool children should minimize requirements for literacy, orthographic knowledge, and complex verbal comprehension while preserving continuous goal-directed behavior. Children at the prewriting stage can complete structured symbol drawing, path tracing, and figure copying tasks while generating quantifiable temporal, spatial, and kinematic data [22,44]. These activities impose limited language demands, present transparent goals, and produce continuous responses. They therefore allow attentional regulation to be expressed through observable movement processes without requiring mature handwriting skills.

Distinct attentional functions should be manipulated through clearly specified task structures. A salient starting cue can vary readiness and orienting demands. Increasing task duration can increase sustained-attention and goal-maintenance demands. Visually similar irrelevant branches or symbols can manipulate selective-attention demands, whereas a change in the relevant target or rule can require reorientation and executive control. These manipulations provide theoretically defined attentional demands against which changes in PGAM regulatory dynamics can be tested [39,61].

Interpretability depends on ensuring that experimental conditions differ primarily in the attentional demand of interest. Sustained attention should be examined by varying task length or duration while maintaining similar path geometry and motor precision. Selective attention should be examined by retaining the target path while varying the number or similarity of distractors. Reorientation should be examined by preserving the basic motor response while changing the relevant target or rule cue. Fabio et al. [62] reported group-related and load-related differences in a figure-tracing dual-task paradigm, providing indirect evidence that graphomotor performance can be sensitive to task demands. The study did not directly validate attention-specific PGAM indicators. Attentional sensitivity should therefore be established through matched experimental contrasts. Performance on a single task is insufficient.

Task parameters should be selected so that changes in task difficulty remain psychologically interpretable. Longer paths increase the duration over which goal-directed behavior must be sustained, whereas distractors increase demands on target selection. Narrower boundaries and greater curvature place greater demands on visual–motor control, while intersections introduce additional route-selection and planning requirements. Manipulating several of these parameters simultaneously can create undifferentiated task difficulty. Structurally matched tasks should therefore vary attentional and motor demands through single-factor or orthogonal contrasts.

An initial PGAM battery could use four matched conditions: a baseline for general graphomotor performance, an extended condition for goal maintenance, a distraction condition for target selection, and a cue-switching condition for reorientation. Matching graphic scale, path structure, and response format would make trajectory differences traceable to defined attentional demands and provide a clear basis for indicator construction.

### 4.2. Constructing Candidate Process Indicators from Trajectory Features

Defined attentional demands provide the theoretical reference for interpreting continuous pen records. PGAM distinguishes the attentional functions engaged by task demands from the regulatory dynamics expressed in graphomotor performance. Together, engagement, maintenance, disruption, and recovery capture complementary temporal dynamics in the unfolding of goal-directed regulation. Candidate indicators are evaluated according to whether their temporal organization varies systematically with theoretically defined attentional demands and remains distinguishable from competing perceptual and motor influences. Indicator construction is therefore theory driven. Raw trajectory signals acquire construct-relevant meaning through their transformation into prespecified behavioral events and process indicators linked to the target regulatory component and its hypothesized behavioral expression. Digital graphomotor research shows that continuous recording preserves timing, spatial control, and online correction beyond the final graphic product [22,33]. This theoretical-to-operational mapping is summarized in Table 3.

PGAM organizes measurement across five levels: basic signals, movement parameters, behavioral events, process indicators, and the resulting regulatory profile. Raw trajectory signals are transformed into movement parameters and temporally localized behavioral events. These events are then integrated into candidate indicators of engagement, maintenance, disruption, and recovery and, if supported by validity evidence, may be combined into a regulatory profile. This hierarchy keeps interpretation traceable from the target construct to observable behavior.

At the process level, engagement can be represented by the latency from task presentation to the first valid movement, the time required to position the pen, the number of aborted starts, and the directional accuracy of the initial stroke. These measures describe how efficiently the child identifies the target and begins an appropriate response. Maintenance can be represented by uninterrupted effective movement, pause structure, local velocity variability, rhythmic stability, and changes across the course of the task. These measures describe the continuity of goal-directed behavior. Disruption can be identified through unexpected pauses, atypical pen lifts, abrupt changes in velocity or direction, path deviations, boundary violations, and movements toward irrelevant targets. Recovery can be characterized by the latency from deviation to correction, the distance traveled before correction, the efficiency of the return path, repeated correction, and the restoration of movement stability after correction. Downing and Caravolas [40] found in school-aged children that selective attention predicted handwriting fluency beyond graphomotor and spelling skills. Research involving ADHD has reported differences in writing-size consistency, non-contact time, speed or pressure variability, and other process measures, although findings vary across tasks and samples [24,49,50]. Kim et al. [39] further distinguished remaining engaged with a target from returning after distraction, providing a developmental basis for distinguishing maintenance and recovery within the organization of goal-directed regulation.

Process indicators acquire meaning from both event location and task demand. Deceleration at a turn, a pause at a decision point, or increased adjustment in a narrow path may be appropriate responses. Velocity, pauses, and deviations should therefore be interpreted relative to path length, curvature, boundary width, and expected decision locations. This contextualization separates graphic and motor complexity from attention-related variation.

Terms such as “unexpected” require an explicit reference. In PGAM, a pause, pen lift, velocity change, direction change, or path deviation should be described as reference-deviant only when it is defined relative to the task structure and a prespecified comparison standard. Task structure includes expected decision points, turns, target changes, correction opportunities, and path boundaries. Comparison standards may include a child’s own baseline condition, low-demand segments within the same task, age- and task-matched reference data, or, in more advanced studies, a simple kinematic model of expected movement. For example, minimum-jerk models may provide a reference for smooth point-to-point movement, whereas curvature-based models may be useful for curved graphomotor patterns [63,64]. These models should be treated as reference tools, not as assumptions that every child’s movement must satisfy.

Pause-based indicators also require explicit thresholds. Writing and handwriting studies have used different pause thresholds, ranging from brief execution-related pauses to pauses of two seconds or longer [65,66]. For this reason, PGAM studies should report how inactivity is defined, such as pen-up time or near-zero movement with pen contact, and should state the threshold used for event detection. Pause duration should be analyzed as a continuous variable whenever possible. If event counts are reported, they should be repeated across prespecified threshold bands, such as brief, intermediate, and long pauses. Candidate indicators should be retained only when the main interpretation remains stable across reasonable threshold choices.

Individual features also support several plausible interpretations. A pause may indicate attentional disengagement or deliberate checking. Reduced velocity may accompany greater attentional demand or limited motor proficiency. Repeated correction may reflect unstable regulation or timely error monitoring. PGAM should therefore focus on patterns across time and across indicators. Task entry, sustained stability, target deviation, and corrective recovery should be examined within the same behavioral sequence. Multidimensional digital handwriting studies show that relations among dynamic features reveal patterns of behavioral organization that are not captured by isolated means or final graphic products [23,27].

Within this proposed hierarchy, basic signals are transformed into movement parameters, movement parameters are organized into behavioral events, and behavioral events are combined into candidate indicators of regulatory dynamics. Indicator meaning would be jointly determined by task demand, event type, temporal position, and pattern configuration. This structure is intended to provide a transparent basis for testing attentional sensitivity and construct validity before any indicator is used in screening. These indicators remain theory-guided candidates requiring further validation, and should be evaluated as temporally organized multivariate patterns, not isolated values.

### 4.3. Data Acquisition for Process Preservation and Measurement Comparability

Data acquisition must preserve the full temporal structure of graphomotor behavior. Digital tablets and smart pens can synchronize coordinates, time, contact, pressure, and task events so that changes in goal-directed regulation can be localized within a continuous behavioral sequence. Raw trajectories should remain intact, with stimulus presentation, first valid movement, distractor onset, deviation, and correction aligned to a common clock. Each process indicator can then be traced to a defined task event and movement segment. Acquisition and quality-control settings should follow predefined PGAM process definitions, with device-specific defaults treated only as implementation parameters.

Process data are comparable only when acquisition conditions remain stable. Writing medium, surface friction, visual feedback, and proprioceptive feedback can systematically alter movement velocity, pressure, and trajectory form in children [43,45]. Device model, stylus characteristics, display scale, active area, sampling frequency, and feedback format should therefore be standardized. Cross-device studies require calibration or bridging measurements to establish correspondence between indicators. Preprocessing should follow prespecified rules for missing samples, unintended contact, and implausible coordinate changes. Filtering should remain sufficiently conservative to preserve brief pauses, directional shifts, and corrective movements that may carry psychologically relevant information.

Consumer devices already support high-resolution graphomotor recording at the prewriting stage [22,23]. The number of features available from a device, however, does not determine measurement quality. Classification performance varies substantially with task structure, sample composition, and analytic procedures [23,27,59]. Data acquisition should prioritize temporally synchronized, clearly defined, and reproducibly computed process records while avoiding unrestricted feature expansion. Only when records are complete and acquisition conditions are comparable can trajectory variation be evaluated as a candidate indicator of attention.

### 4.4. Identification and Establishment of Sensitive Indicators

Candidate graphomotor indicators should first demonstrate stable and reproducible responses to variation in attentional demand. The strongest initial evidence comes from matched within-child comparisons. Task duration, distractor load, and reorientation requirements can be manipulated while path structure and motor demands remain largely constant. Maintenance indicators should change as task duration increases. Selection indicators should respond to greater distractor load. Recovery indicators should capture correction latency and the restoration of stability following a cue-induced change. Fabio et al. [62] reported group-related and load-related differences in a figure-tracing dual-task paradigm, providing indirect evidence of task sensitivity but no direct validation of PGAM.

Experimental sensitivity also requires support from independent measures of attention. Candidate indicators should show theoretically consistent associations with sustained attention, selective attention, structured observation, and parent and teacher ratings. They should also retain explanatory value beyond basic motor ability and visual–motor integration. Hwang et al. [41] found that preschool inattention, manual dexterity, and visual–motor integration predicted different aspects of later handwriting performance. This pattern suggests that attentional, perceptual, and motor factors may make partly distinct contributions to graphomotor outcomes. It does not establish attention specificity for individual trajectory indicators. PGAM studies should therefore assess these developmental domains concurrently and use competing models to identify trajectory features that primarily reflect attentional regulation.

Candidate indicators must also remain stable across trials, measurement occasions, and independent samples. Hong et al. [54] reported substantial variation in the test–retest stability of preschool handwriting measures. In a primary-school sample, Lu et al. [55] found that the factor structure and factor loadings of a handwriting-legibility scale were comparable across grades, but intercept differences between some grade groups indicated potential limitations in direct mean-score comparisons. Establishing a sensitive indicator therefore requires evidence of responsiveness to controlled attentional demands, convergence with external attention criteria, discrimination from motor influences, and reproducibility across measurements and samples. Only indicators meeting these criteria should be retained within PGAM.

## 5. Potential Role of PGAM in Early Screening and Developmental Support

The central translational question is whether PGAM process indicators improve early screening decisions. PGAM is designed as a complementary layer within multimethod assessment: it can characterize how regulation unfolds, guide targeted reassessment, and sharpen referral decisions when interpreted alongside developmental level and evidence from multiple sources. Formal validation should establish the reliability, construct meaning, incremental value, and decision accuracy required for screening use.

### 5.1. A Process-Based Complement to Multimethod Early Screening

Preschool attention varies across tasks, environments, and observers, so each method contributes a distinct view. Parent and teacher ratings capture behavior across home and educational settings. In a large preschool ADHD sample, agreement between informants was low, potentially reflecting differences in observation context [12]. Continuous performance tests standardize accuracy, reaction time, and variability [17], while objective activity measures characterize motor behavior [18]. PGAM contributes a time-resolved record of how goal-directed regulation unfolds during continuous action. Integrating these sources aligns assessment with the evidence that childhood attention is best understood across methods, informants, and settings [11].

This process perspective differs from methods that primarily summarize performance across trials or observation periods. Continuous trajectory data provide a basis for characterizing how goal-directed regulation is established, maintained, disrupted, and restored over the course of task performance. Informant ratings summarize behavior across settings, continuous performance tests characterize accuracy and response timing across discrete trials, and activity measures characterize overall motor behavior. PGAM instead differentiates engagement, maintenance, disruption, and recovery, creating a basis for distinguishing regulatory profiles among children with similar overall performance.

The maintenance indicators proposed in PGAM are most directly related to the intra-individual variability tradition in attention research, but they should not be treated simply as pen-based equivalents of reaction-time variability. Variability in contact duration, pause structure, velocity, pressure, path stability, and correction timing may extend this tradition from the magnitude of trial-to-trial variability to the temporal organization of instability within continuous goal-directed action. Graphomotor trajectories can potentially characterize when instability emerges, how long it persists, whether it clusters at specific task locations or demands, and how efficiently stable performance is restored after disruption. Because graphomotor behavior continuously couples perception, action, and online correction, the same trajectory can also indicate where a fluctuation occurs in relation to task structure and what behavioral adjustment follows it. These potential advantages, however, cannot be assumed simply because the measures are derived from continuous pen movement. Their incremental value must be tested directly against established variability indices from attention tasks of comparable duration, including ex-Gaussian τ and, where reliably estimable, diffusion-model parameters [15,16].

The central comparison is therefore not whether PGAM produces another measure of behavioral variability, but whether the temporal organization of graphomotor instability provides information that is not fully represented by conventional response-time variability. Incremental value should also be examined alongside informant ratings, structured observation, and standardized attention tasks. Differences across methods can clarify context: a trajectory pattern that emerges only during prolonged, distracting, or rule-switching conditions may identify sensitivity to a specific attentional demand and guide reassessment. Developmental calibration then determines whether that pattern carries screening relevance.

### 5.2. Developmentally Calibrated Interpretation of Process Profiles

Graphomotor behavior changes rapidly as motor planning, manual dexterity, visual–motor integration, rule maintenance, and self-monitoring develop. A delayed start, velocity fluctuation, or repeated correction may therefore reflect different mechanisms. Initiation latency may reflect differences in task engagement, target localization, or motor preparation. Pauses and velocity changes may similarly reflect regulatory instability or appropriate adjustment to path complexity. These measures should therefore be interpreted in relation to age, task demands, and baseline graphomotor proficiency.

Available evidence supports this developmental constraint. Hong et al. [54] found substantial variation in the test–retest stability of handwriting measures among children aged five to six years, indicating that some observed features are sensitive to task conditions and measurement occasion. Hwang et al. [41] showed that inattention, manual dexterity, and visual–motor integration predicted different aspects of subsequent handwriting performance from kindergarten to first grade. These findings suggest that attentional, perceptual, and motor factors contribute to graphomotor behavior through partly distinguishable pathways. Research in primary-school children further indicates that the interpretation of a handwriting construct may vary across age groups, although these findings do not directly validate preschool PGAM measures [55].

A defensible interpretation combines age-referenced norms, developmental characterization, and within-child comparison. Narrow age bands establish the relevant peer reference. Manual dexterity, visual–motor integration, and general graphomotor proficiency help separate likely sources of variation. Matched conditions test whether an indicator responds selectively to duration, distractor load, or reorientation demand, while repeated measurements establish stability across trials and occasions.

Process profiles warrant greater weight in retesting or referral when they consistently depart from age expectations, recur across measurements, and vary with the target attentional demand as predicted. Developmental calibration converts fine-grained trajectory variation into defensible individual-level information for tiered decisions.

### 5.3. Integration into Tiered Screening and Referral

Developmentally calibrated PGAM profiles can be embedded within a progressive sequence of broad screening, targeted reassessment, and comprehensive professional evaluation. This tiered structure increases interpretive confidence as evidence accumulates and reduces the risk that transient performance changes associated with task unfamiliarity, fatigue, misunderstanding, or situational variation are overinterpreted. At the broad-screening stage, brief, low-language, structurally matched tasks can identify process profiles that deviate from age-referenced expectations. The aim is risk stratification, not stand-alone identification. As such, PGAM should be interpreted within a broader clinical or developmental context. At low base rates, even apparently strong sensitivity and specificity can still yield a substantial proportion of false-positive classifications, so an elevated screening profile should prompt reassessment, not be interpreted as evidence of disorder. Decision rules should distinguish routine monitoring, targeted reassessment, and referral while incorporating uncertainty surrounding isolated trajectory events. Task design should prioritize comprehension and completion while preserving sensitivity to attentional demands and maintaining comparable perceptual and motor requirements across conditions. Population-level decision thresholds should also account for the prevalence of the target risk state and the relative consequences of false-positive and false-negative classifications. PGAM-based screening should therefore inform staged decision-making, with diagnostic classification reserved for comprehensive professional evaluation.

Targeted reassessment examines whether an initially atypical profile is stable and specific to the hypothesized attentional demand. Additional trials, repeated measurement occasions, and matched manipulations of task duration, distraction, and reorientation can determine whether the pattern recurs and changes as theoretically predicted. Recurrent problems in engagement or maintenance, repeated disruption under distraction, or inefficient recovery provide stronger evidence for subsequent evaluation than an isolated trajectory anomaly.

Persistent atypical process profiles should be interpreted alongside parent and teacher ratings, structured observation, standardized attention tasks, developmental history, and functional performance. Professional evaluation of childhood attention problems requires evidence from multiple informants, settings, and domains, and should not rely on any isolated objective measure [11,67]. Within this multimethod framework, PGAM provides process-level information about how regulation is organized and where difficulty is concentrated, including task engagement, goal maintenance, susceptibility to disruption, and recovery following deviation. Such information can sharpen the focus of follow-up assessment and the rationale for referral, while diagnostic conclusions remain grounded in comprehensive professional evaluation.

### 5.4. Process-Informed Developmental Support and Monitoring

Process differences identified through tiered assessment may also generate hypotheses for developmental support. Children with similar overall completion scores may show distinct difficulties in task engagement, sustained maintenance, resistance to distraction, or recovery after deviation. Process information can therefore decompose a broad indication of attentional risk into more specific and observable components of goal-directed behavior.

Delayed task initiation may prompt closer examination of goal comprehension, starting-point localization, and action preparation. Pronounced variability during an extended task may indicate that goal maintenance warrants further observation. Frequent movements toward irrelevant paths or symbols may suggest difficulty selecting task-relevant information or resisting distraction. Prolonged failure to return following deviation may implicate reorientation and online correction. Selective allocation of attention to task-relevant information is developmentally consequential for learning in children aged three to five years [61]. Attention maintenance and the capacity to return to a target after distraction also follow distinguishable developmental trajectories between 3.5 and 6 years of age [39]. These findings provide a developmental rationale for examining maintenance and recovery separately.

Process profiles may also generate hypotheses for developmental support. Engagement difficulty directs attention to goal cues and task-entry structure. Maintenance instability motivates comparison across task duration and interim guidance. Distractor sensitivity focuses evaluation on target salience and irrelevant information, while delayed recovery prompts testing of reorientation cues and self-checking. These mappings turn PGAM profiles into testable support hypotheses for future intervention research.

Repeated PGAM assessment may also help characterize changes associated with natural development or targeted support. Initiation latency, local variability, deviation frequency, and recovery efficiency could provide more differentiated information than a single total score. Monitoring applications, however, require sufficient test–retest reliability and sensitivity to meaningful change. They must also distinguish genuine developmental change from measurement error and practice effects. The variation in indicator stability reported by Hong et al. [54] underscores the need for reliability evidence before longitudinal interpretation.

PGAM’s immediate contribution to developmental support is structured process information for defining reassessment conditions, observational priorities, and monitoring targets. Longitudinal and intervention studies can then determine whether candidate indicators track meaningful developmental change and intervention response.

## 6. Discussion

PGAM reframes preschool attention assessment from static task outcomes toward the temporal organization of goal-directed regulation during ongoing action. Current evidence supports the technical feasibility and theoretical plausibility of continuous graphomotor measurement, but does not yet establish attention-specific or screening-valid indicators. The principal contribution of the framework therefore lies not in a new set of trajectory features, but in a traceable measurement logic linking controlled attentional demands, temporally localized graphomotor events, regulatory dynamics, and multimethod validation. The scientific value of this approach will depend on whether the resulting process measures are reliable across development, support attention-related interpretation beyond competing motor and perceptual influences, and add meaningful value to multimethod screening decisions.

### 6.1. Conceptual and Technical Contributions and Interpretive Scope

The proposed conceptual contribution of PGAM is not the product–process distinction itself. This distinction is already well established in digitized handwriting research. Rather, PGAM applies process information to a distinct measurement problem concerning how attention-related regulation may become observable during preschool graphomotor tasks. Accuracy, completion time, and final graphic quality summarize performance, but reveal little about when regulation changes during task performance or how such changes unfold over time. Continuous graphomotor activity provides a behavioral record in which these changes can be temporally localized and tested against task demands. Children at the prewriting stage can complete structured symbol-drawing, path-tracing, and figure-copying tasks while producing quantifiable temporal, spatial, and kinematic data [22,44]. These activities may therefore provide a developmentally appropriate measurement context with limited language demands, clear goals, and continuous responses.

The proposed technical contribution of PGAM is not a new set of trajectory indicators, but a traceable measurement chain connecting task-defined attentional demands, observable graphomotor events, and their proposed cognitive interpretation. Matched task conditions can vary attentional demands while keeping graphic and motor requirements as comparable as possible. Continuous trajectories would then be segmented into task-relevant behavioral events and integrated according to their timing, duration, sequence, and recovery structure. These temporally organized events provide candidate measures of regulatory dynamics whose interpretation can be tested against theoretically specified attentional demands. Synchronization between task events and graphomotor records would allow each interpretation to be traced to a defined condition and movement segment. Previous digital graphomotor studies show that continuous acquisition preserves movement timing, spatial control, and online correction that cannot be recovered from the final graphic product [22,26,33]. PGAM extends this capability by integrating experimental manipulation, event-level measurement, and cognitive interpretation within the same measurement architecture.

PGAM also offers a principled framework for organizing high-dimensional trajectory data by grounding indicator construction in theory. Raw device signals acquire psychological meaning only through theory-guided transformation into defined behavioral events and process indicators linked to the target regulatory component and its hypothesized behavioral expression. Velocity, pauses, deviations, and corrections therefore derive their interpretive value from their temporal and spatial context and from the task conditions under which they occur. Temporally organized behavioral patterns may consequently provide a more informative unit of analysis than isolated trajectory features, while reducing post hoc interpretation and improving reproducibility.

A central interpretive challenge is that graphomotor behavior is inherently multicausal. Performance reflects visual encoding, spatial organization, motor planning, manual dexterity, visual–motor integration, and prior task experience. Studies involving children with ADHD suggest that group differences may be expressed through longer in-air time, greater movement variability, or reduced fluency, but none of these features is intrinsically specific to attention [24,49,50]. Hwang et al. [41] similarly found that inattention, manual dexterity, and visual–motor integration predicted different aspects of subsequent handwriting performance. Because attentional and motor influences converge on the same graphomotor output channel, motor variation directly complicates construct attribution and should be explicitly incorporated into PGAM validation. PGAM therefore treats perceptual and motor contributions as part of the construct-identification problem. An attention-related interpretation requires evidence that candidate indicators respond systematically to controlled attentional demands and converge with independent attention criteria, while remaining distinguishable from motor coordination, visual–motor integration, and baseline graphomotor proficiency. Statistical adjustment alone is insufficient to establish this distinction.

### 6.2. Validation Pathway from Process Sensitivity to Screening Utility

The development of PGAM from a conceptual and technical framework into a screening instrument requires a coherent validity argument linking experimental responses, measurement stability, construct interpretation, and decision utility. Validity pertains to the interpretation and proposed use of scores and must be evaluated against the intended inferences and decisions. The Standards for Educational and Psychological Testing and related validity theory emphasize that group differences, correlations, or classification accuracy cannot independently establish such an argument [68,69,70].

Initial evidence should establish that candidate indicators show theoretically specific sensitivity to the intended attentional demand. When graphic structure and motor requirements are held broadly constant, longer task duration should preferentially affect maintenance indicators, greater distractor load should preferentially affect disruption-related indicators, and changes in target or rule cues should affect reorientation and recovery-related indicators. Fabio et al. [62] reported group- and load-related differences in a figure-tracing dual-task paradigm, providing indirect evidence of task sensitivity. Such findings demonstrate responsiveness to task demands but do not yet distinguish attentional regulation from general cognitive load or altered motor control. Stronger evidence would require theoretically predicted changes to occur under the corresponding attentional manipulation while remaining comparatively limited under matched control conditions that closely match perceptual and motor demands.

Process sensitivity can support individual assessment only when it is measured reliably. Candidate indicators require adequate within-session or cross-trial consistency, test–retest reliability, and structural stability. Hong et al. [54] found substantial variation in the test–retest stability of handwriting measures among children aged five to six years, demonstrating that technically computable features are not necessarily suitable indicators of stable individual differences. Indicator structure should first be established within narrow age bands and then tested for measurement equivalence across age groups, task versions, and devices. Comparisons across children or measurement occasions are interpretable only when scores retain sufficiently similar meanings across the relevant conditions.

Construct interpretation requires convergence and discrimination. PGAM indicators should converge with independent measures of attention, including sustained and selective attention measures, behavioral observations, and informant ratings. They should also remain distinguishable from manual dexterity, visual–motor integration, processing speed, and baseline graphomotor skill. Because attentional and motor influences are expressed through the same graphomotor output channel, concurrent standardized measures of motor coordination and visual–motor integration, together with baseline graphomotor proficiency, should be incorporated into construct-validation analyses. Attention-related interpretations of PGAM indicators should be tested against plausible motor, perceptual, and developmental alternatives through explicit model comparison, supplemented by statistical adjustment where appropriate. An attention-related interpretation is supported when candidate indicators vary as predicted with attentional demands, converge with independent attention criteria, and retain explanatory value beyond these competing sources of variation.

Incremental validity asks whether PGAM provides information beyond established assessment methods. For maintenance indicators, this test should be specified in direct relation to established measures of intra-individual variability. PGAM studies should compare pen-based variability with response-time variability from continuous performance tasks of comparable duration. Hierarchical or competing-model analyses should first include established CPT measures, such as accuracy, mean reaction time, and ex-Gaussian τ, with diffusion-model parameters included where the task structure permits reliable estimation [15,16]. Pen-based indicators should then be added to determine whether the temporal organization of graphomotor instability provides incremental information beyond conventional response-time variability. Of particular interest are when instability emerges, how long it persists, whether fluctuations cluster at specific task-relevant locations or attentional demands, what corrective behavior follows a deviation, and how efficiently stable performance is restored. Incremental validity should be evaluated primarily against independent attention-related criteria, including behavioral observations, parent or teacher ratings, and later attention-related functioning, with broader functional outcomes providing additional evidence where appropriate.

The critical comparison is therefore not whether PGAM produces another measure of behavioral variability, but whether continuous graphomotor dynamics capture the temporal organization and resolution of regulatory instability beyond that represented by established CPT indices. Incremental prediction would support the unique contribution of pen-based variability to attention assessment. Without such gain, its value would lie primarily in offering an alternative behavioral context for measuring attentional variation. More broadly, the contribution of PGAM should be evaluated alongside ratings, observation, and conventional attention tasks, especially among children with similar overall performance but different patterns of engagement, maintenance, disruption, and recovery. Such distinctions could refine retesting conditions, problem formulation, and referral decisions.

Screening utility should be evaluated in representative populations using sensitivity, specificity, predictive values, and calibration, interpreted against prevalence and the consequences of false decisions. This requirement is especially important when base rates are low. Given an ADHD prevalence of 4.2–5.0% [4], a hypothetical screening tool with sensitivity and specificity of 0.90 would yield a positive predictive value of only approximately 28–32%. This calculation illustrates how base rates shape the practical value of screening performance. Screening measures with strong sensitivity and specificity are most informative when integrated into monitoring, reassessment, and referral pathways. Accordingly, PGAM should be evaluated by its incremental contribution to multimethod decision making beyond established measures [17,18]. Longitudinal studies can then determine whether early process profiles predict subsequent attentional functioning, functional outcomes, and professional assessment findings.

### 6.3. Remaining Evidence Gaps and Validation Priorities

The current evidence supports the technical feasibility and theoretical plausibility of pen-based graphomotor assessment, but it remains insufficient to establish PGAM as a validated approach to preschool attention screening. Research at the preliteracy stage shows that young children can complete tablet-based graphomotor tasks and generate analyzable process data [22]. Evidence linking attention to graphomotor performance, however, is derived primarily from school-aged children, adolescents, and clinical ADHD samples [40,49,50]. These studies identify potentially relevant associations between attention-related functioning and handwriting fluency, variability, or quality, but they do not demonstrate that the same indicators provide attention-specific information in representative preschool populations. The available literature therefore supports separate components of the proposed measurement pathway, with a complete validation chain yet to be established.

The principal evidence gap is not the absence of potentially informative trajectory features, but the lack of evidence connecting attentional demands, temporally localized graphomotor responses, and valid individual-level interpretation. A defensible PGAM measure must demonstrate that controlled changes in attentional demand produce theoretically interpretable changes in graphomotor behavior and that these changes can be distinguished from variation in visual analysis, visual–motor integration, manual dexterity, motor planning, task comprehension, motivation, and prior graphomotor experience. Existing findings indicate that motor and visual–motor abilities make substantial and partly distinct contributions to handwriting outcomes [41,53]. The proposed dimensions of engagement, maintenance, disruption, and recovery therefore require direct empirical examination of their empirical coherence, temporal organization, and reproducibility across tasks.

Developmentally sensitive validation is equally important. Graphomotor performance changes rapidly during the preschool years as children acquire greater motor control, symbolic knowledge, task familiarity, and experience with writing-related activities. Features that are informative within one age group may differ in their distribution or psychological meaning in another. Validation studies should therefore use sufficiently narrow developmental bands, establish age-appropriate reference distributions, and evaluate test–retest reliability, measurement invariance, device equivalence, and reproducibility across task versions. Studies comparing writing media and feedback conditions indicate that recording medium and task configuration can influence movement characteristics in children [43,45]. Multisite and cross-cultural studies are consequently needed to determine which indicators generalize across writing systems, educational practices, devices, and cultural contexts.

Predictive modeling introduces additional methodological challenges because digital trajectories can generate many correlated features, increasing the risk of overfitting and unstable feature selection. Computational studies demonstrate the discriminative potential of graphomotor data in handwriting impairment and developmental variation [23,27,59]. Extending this potential to attentional regulation requires construct-specific validation. Future PGAM studies should therefore use adequately powered samples, prespecified analytic pipelines, principled feature reduction, and independent external validation. Model evaluation should consider calibration, subgroup performance, and uncertainty in addition to overall discrimination. Predictive performance must also be supported by appropriate construct and criterion validity.

The practical value of PGAM will be determined by the information it adds to existing assessment. Studies should test incremental validity over parent and teacher reports, behavioral observation, established attention tasks, and motor-development measures, then evaluate prediction, subgroup fairness, calibration, and decision consequences in representative samples. The present conceptual and critical narrative review supplies the theory-guided framework for that program: PGAM defines the tasks, process indicators, competing explanations, and validation criteria needed to move from continuous trajectories to interpretable screening evidence.

### 6.4. Generalizability and Responsible Translation

Responsible translation begins with developmental and contextual calibration. Systematic reviews indicate that relatively younger children within the same school cohort are more likely to receive ADHD diagnoses or medication, while concerns regarding overdiagnosis are particularly relevant to milder presentations [71,72]. These findings illustrate how developmental reference points can influence identification and reinforce the need for age-calibrated, staged, and multimethod interpretation. Age and cultural experience may also affect task comprehension and movement strategy. Familiarity with digital devices, exposure to graphic activities, and opportunities for pen use vary across educational settings, family resources, and cultural environments. The same task may therefore impose different cognitive or motor demands across groups. Age-referenced norms should be established in representative samples using narrow developmental bands, while cross-linguistic and cross-cultural applications require renewed evaluation of task familiarity, indicator structure, and decision thresholds. Fair measurement requires evidence that observed group differences arise from the target process and are not attributable to unequal educational opportunities or digital experience [68].

Generalizability also depends on measurement comparability across devices and recording environments. Writing medium, surface friction, visual feedback, and proprioceptive feedback can alter movement velocity, pressure, and trajectory form [43,45]. Tablets, styli, and sampling systems therefore require calibration for spatial scale, temporal precision, pressure range, and feedback characteristics. Device replacement or cross-platform implementation should be accompanied by bridging studies that determine whether indicator distributions and decision thresholds remain comparable across systems.

Responsible use further requires that PGAM-derived information remain interpretable and traceable. Process indicators should be linked to clearly specified task conditions, behavioral events, and analytical procedures, with uncertainty represented explicitly. Transparent task rules, indicator definitions, preprocessing procedures, modeling strategies, and external validation allow professionals to evaluate the basis of each estimate and support replication across studies. Graphomotor trajectories also contain temporally detailed behavioral information, making purpose limitation and data minimization important considerations. Research and implementation require informed parental consent, age-appropriate information and assent procedures for children, and explicit policies governing data access, retention, and secondary use.

The boundaries of decision use should remain equally explicit. PGAM may inform continued observation, targeted reassessment, developmental monitoring, and professional referral. Clinical diagnosis, educational placement, and resource allocation should continue to depend on comprehensive professional evaluation incorporating multiple informants and settings, developmental history, functional impairment, and appropriate clinical judgment [11,67]. Translation into early screening will therefore depend on whether process indicators preserve their meaning across development, populations, contexts, and devices and provide useful information within multimethod decisions without increasing avoidable misclassification or inequity.

## 7. Conclusions

This conceptual and critical narrative review develops pen-based graphomotor attention measurement (PGAM) as a hypothesis-driven approach for studying the continuous regulation of goal-directed graphomotor behavior in preschool children. By examining how engagement, maintenance, disruption, and recovery might be represented during familiar graphomotor tasks, PGAM specifies candidate moment-to-moment regulatory patterns that are not adequately captured by endpoint measures such as accuracy, completion time, or graphic quality.

PGAM is a proposed measurement-development framework grounded in a developmental account of how attentional regulation may be expressed during graphomotor activity. The account provides the explanatory bridge between established attention theory and observable movement organization. PGAM supplies the measurement architecture by mapping this relation onto task manipulations, behavioral events, candidate trajectory indicators, and explicit validation tests.

PGAM is designed to complement multimethod assessment by adding process-level information about how regulation unfolds during continuous goal-directed activity. Its validation agenda centers on reliability, developmental reference distributions, construct and incremental validity, screening accuracy, fairness, and implementation value in representative preschool samples. Evidence meeting these standards would position PGAM to strengthen early screening, targeted reassessment, and developmental monitoring for attention-related difficulties.

## Figures and Tables

**Table 1:** Positioning of PGAM Relative to Existing Handwriting and Graphomotor Assessment Approaches.

Approach	Primary Focus	Process Information	Relation to PGAM
Digitizer-based process analysis [25,26,33]	Handwriting production	Timing, velocity, pauses, pressure, trajectory characteristics	Provides established graphomotor process variables
Eye and Pen [28]	Visual–writing coordination	Temporal coordination between gaze and writing	Demonstrates time-resolved analysis across perceptual and motor activity
Digital BHK and related approaches [23,27]	Handwriting quality and production dynamics	Product scores combined with dynamic measures	Extends handwriting assessment from outcome to production process
Tablet-based feature sets [23,29,30]	Digital characterization of graphomotor performance	Temporal, kinematic, pressure, and spatial features	Provides multidimensional process features applicable to graphomotor analysis
Handwriting Proficiency Screening Questionnaire (HPSQ)/Handwriting Proficiency Screening Questionnaire for Children (HPSQ-C) [31,32]	Perceived handwriting difficulty	Teacher report or child self-report	Provides complementary functional information without continuous process data
Pen-based Graphomotor Attention Measurement (PGAM)	Attentional regulation during preschool graphomotor activity	Established process signals organized as temporally localized behavioral events	Links graphomotor events to theoretically specified attentional processes

**Table 3:** Theoretical and Operational Mapping of PGAM Regulatory Dynamics.

PGAM Regulatory Component	Primary Attentional Functions	Regulatory Definition	Recommended Task Manipulation	Candidate Graphomotor Indicators
Engagement	Alerting; orienting	Establishment of functional alignment between the task goal and an appropriate initial action	Salient starting cue and clearly specified target under matched motor requirements	Initiation latency; initial hesitation; aborted starts; initial directional accuracy; early movement organization
Maintenance	Sustained attention; goal maintenance	Stability and continuity of goal-directed regulation over time	Increased task duration or repeated structurally matched paths with comparable graphic and motor demands	Continuous effective-movement duration; pause distribution; local velocity variability; rhythmic stability; within-task drift
Disruption	Selective attention; executive control under distraction or conflict	Transient destabilization of goal–behavior coordination	Matched distractor load, competing branches or symbols, or controlled rule interference	Reference-deviant pauses or pen lifts; abrupt velocity or directional changes; path deviation; boundary crossings; movements toward irrelevant targets
Recovery	Reorienting; executive control	Re-establishment of stable goal-directed regulation following interruption or deviation	Controlled interruption, target or rule change, or detectable deviation	Correction latency; distance before correction; return-path efficiency; repeated correction; restoration of movement stability

Note: Engagement, maintenance, disruption, and recovery are dynamic regulatory components with many-to-many links to attentional functions. Candidate indicators require validation against competing perceptual, motor, and developmental explanations. PGAM = pen-based graphomotor attention measurement.

## Data Availability

No new data were created or analyzed in this study. Data sharing is not applicable to this article.
